# Prevalence, risks factors, and control of hypertension in Guinean older adults in 2021: a cross-sectional survey

**DOI:** 10.1186/s12889-024-18936-6

**Published:** 2024-06-07

**Authors:** Alioune Camara, Alpha Koné, Thierno Mamadou Millimono, Abdoulaye Sow, Amadou Kaké, Pierre-Marie Preux, Mamadou Dadhi Balde, Pierre Jesus

**Affiliations:** 1 Department of Public Health, Faculty of Sciences and Health Technics, Gamal Abdel Nasser University of Conakry, Conakry, BP: 1017, CP:030 Guinea; 2 African Center of Excellence for the Prevention and Control of Communicable Diseases, Gamal Abdel Nasser University of Conakry, Conakry, Guinea; 3 Department of Cardiology, Faculty of Sciences and Health Technics, Gamal Abdel Nasser University of Conakry, Conakry, Guinea; 4 EpiMaCT - Epidemiology of Chronic Diseases in Tropical Zone, Institute of Epidemiology and Tropical Neurology, Inserm U1094, IRD U270, CHU Limoges, Univ. Limoges, Limoges, France; 5 National Program of Prevention and Control of Non-Communicable Diseases, Ministry of Health Public Hygiene, Conakry, Guinea

**Keywords:** Prevalence, Arterial hypertension, Elderly, Associated factors, Guinea

## Abstract

**Background:**

The incidence of arterial hypertension increases with the aging of the population, but its magnitude remains insufficiently assessed. The aim of this study was to investigate the prevalence of hypertension and associated factors in elderly people in Guinea.

**Methods:**

Data were obtained from a cross-sectional general population survey, conducted among people aged ≥ 60 years. A stratified enumeration area random sample survey was conducted in the four natural regions of Guinea from February to April 2021. This study included an interview on sociodemographic data, and a clinical examination. Hypertension was defined as systolic blood pressure ≥ 140mmHg and/or diastolic blood pressure ≥ 90mmHg or previous diagnosis of hypertension (with or without antihypertensive medication). Hypertension control was defined as blood pressure below 140/90 mmHg during treatment. Age-standardized prevalence was calculated, and logistic regression was used to examine factors associated with hypertension.

**Results:**

A total of 1698 adults (1079 men, mean age: 71.6 ± 9.4 years) had at least two blood pressure measurements. The standardized prevalence of hypertension was 61.4% [95% CI: 61.3–61.6], ranging from 52% in Middle Guinea to 67% in Upper Guinea, and was higher in women (65.2%: 65.0-65.4) than in men (59.1%:58.9–59.3). Among those with hypertension, 46.7% were unaware of their condition before the survey and 49.6% were on treatment and only 18.5% had controlled hypertension. Whatever the residence (rural or urban), increasing age, being unmarried, working as a trader or functionary, jobless, living in upper Guinea, low monthly income, intake of extra salt, known diabetic, overweight, and obesity increased the risk of hypertension. In urban area, female sex (AOR: 1.14: 1.12–1.17), living in lower Guinea (AOR: 3.08: 2.97–3.20), being Maninka (AOR: 1.26: 1.21–1.31), being Nguerze (AOR: 1.71: 1.63–1.81) increased the risk of hypertension, but living in forest Guinea (AOR: 0.88: 0.83–0.93), being Soussou (AOR: 0.88: 0.85–0.92) decreased the risk. In rural area, living in forest Guinea (AOR: 2.14: 2.03–2.26), being Soussou (AOR: 1.14: 1.12–1.17) increased the risk of hypertension, but female sex (AOR: 0.96: 0.94–0.98), living in lower Guinea (AOR: 0.87: 0.85–0.89), being Maninka (AOR: 0.94: 0.92–0.97), being Nguerze (AOR: 0.50: 0.47–0.52) decreased the risk.

**Conclusion:**

Hypertension is a major problem in the elderly population in Guinea, and the level of treatment and control in elderly with known hypertension is inadequate. The place of hypertension among cardiovascular diseases and the identification of associated factors underlines the need to develop innovative approaches to control this major risk factor.

## Introduction

Nearly a quarter (23%) of the global burden of disease is due to disability in older people, of which cardiovascular disease (CVD) is the largest contributor [1]. Hypertension is the leading cause of mortality and morbidity from CVD, particularly in the elderly [2, 3]. The prevalence of hypertension is gradually increasing worldwide due to an aging population [3, 4]. According to the United Nations, the number of people aged 65 years and older is expected to more than double by 2035, from 608 million in 2015 to 1.2 billion in 2035 [5].

Hypertension in the elderly is associated with adverse cardiovascular outcomes including coronary heart failure, stroke, myocardial infarction and death [6, 7]. The goal of individualized hypertension management in older adults is to improve overall health outcomes and quality of life, while minimizing the risk of adverse events from antihypertensive medications [6, 8]. It is estimated that by 2025, nearly three-quarters of the hypertensive population will live in developing countries, particularly in sub-Saharan Africa [9]. Some surveys of hypertension in Africa, may use a different age range to define older adults [10–12]. The findings indicate that hypertension manifests at an earlier age within the black African population. However, its prevalence steadily rises with advancing age, consequently predisposing individuals to a spectrum of cardiovascular diseases [13]. Hypertension is the leading risk for death in Africa, accounting for 10% of all deaths on the continent in 2016 [14]. Systematic analysis suggest that the prevalence of hypertension is increasing in Africa and that many hypertensive patients are unaware of their condition [15, 16]. The relatively high prevalence of hypertension in Africa is associated with population growth and aging, increasing urbanization, rural-urban migration, and increasing adoption of a western lifestyles, including tobacco and alcohol use [17, 18].

In low and middle-income countries, the prevalence of hypertension in people over 50 years of age is 52.9%; range 32.3% in India to 77.9% in South Africa [19]. It is important to learn more about the epidemiology of hypertension in older Africans. In Guinea in 2009, the results of a population-based STEPS survey reported a prevalence of hypertension of 29.9% [20]. This prevalence increased with age to attend 62.5% in the 44–64 years age group. Furthermore, in Guinea, there is a notable scarcity of data regarding hypertension among individuals aged 60 and above, compared to the available data for younger adults. Guinea, like 26 other countries, has benefited from support from WHO in 2010, through an approach known as WHO-PEN, which aims to decentralize the management of non-communicable diseases, including hypertension, to the primary health care level [21]. Services include screening and diagnosis, treatment, lifestyle change, patient education, and disease self-management.

To our knowledge, hypertension data from large elderly populations have never been systematically studied in Guinea. The aim of this study was to investigate the prevalence of hypertension in a large Guinean elderly population and to identify associated risk factors.

## Methods

### Study setting and duration

**Fig. 1:**
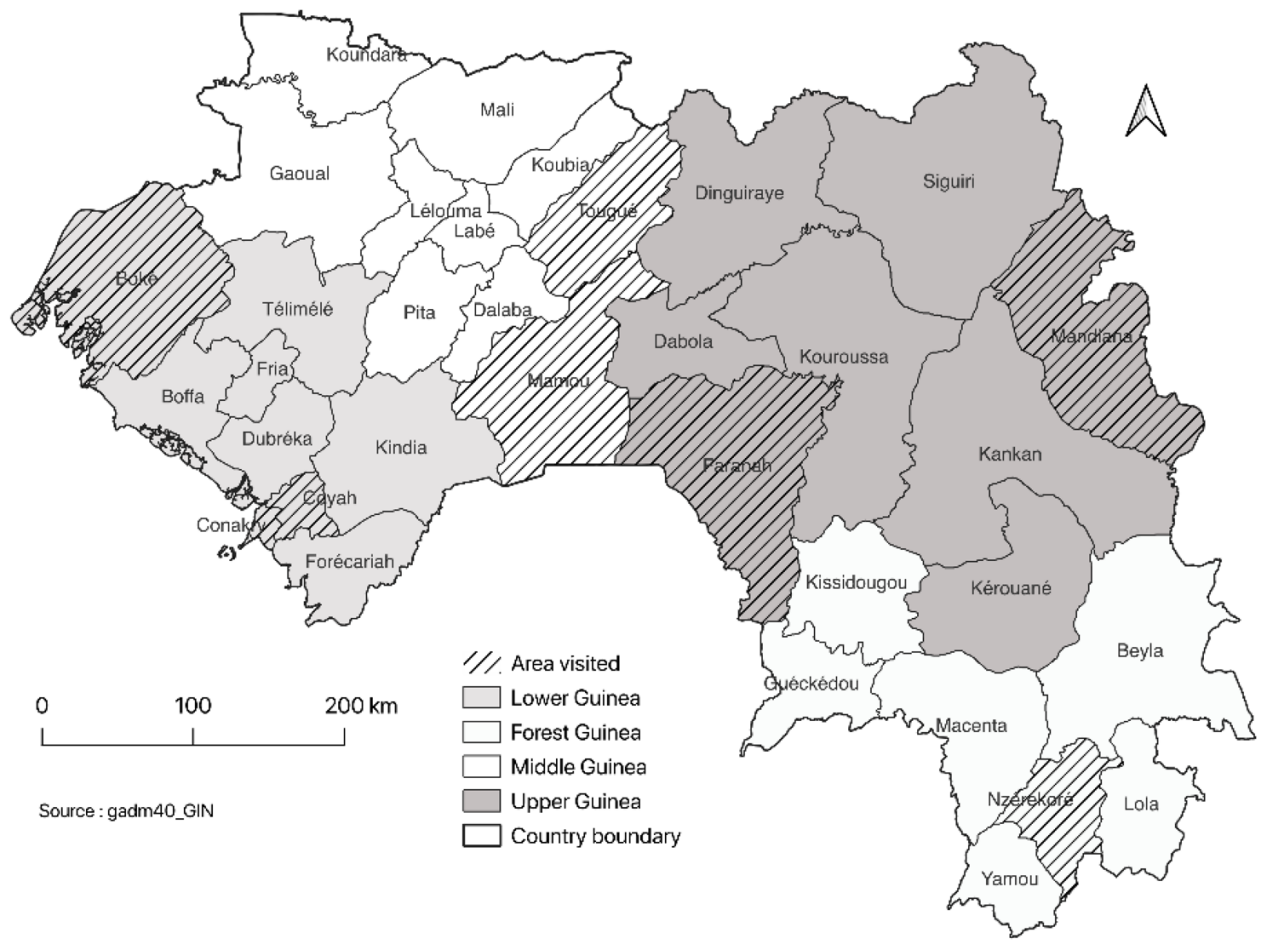
Map of Guinea: Identification of the four regions and the eight areas of collection in 2021

Guinea is a West African country characterized by a tropical climate. Guinea is divided into four natural regions and eight administrative regions (Conakry, Kindia, Boké, Mamou, Labé, Faranah, Kankan and Nzérékoré). Its main economic activities are agriculture, the craft industry, and informal trade. According to the projections of the National Statistical Direction, the population of Guinea in 2021 was 12 907 395 people, including 679, 352 adults aged 60 and over, with 8,173,792 in rural areas and 4,733,603 in urban areas. Each region is further subdivided into districts and the National Statistical Direction enumeration area each district into sectors. An enumeration area was defined as a neighbourhood in an urban area and a locality in a rural area. The map depicting the identification of the four regions and eight collection zones in 2021 is presented in Fig. 1.

### Study design

This study was a national observational, cross-sectional, and analytic study conducted from February to April 2021 in the 8 administrative regions of Guinea.

### Study population and eligibility criteria

The study population for this research consisted of community members aged 60 years and older who had not been hospitalized and had resided in Guinea for at least six months prior to the survey date. Prior to study inclusion, written informed consent (or fingerprints of those who were not able to write) was obtained from all participants.

### Sampling methods

A sample of eligible participants was assembled using the sampling frame. Age was obtained from official documents, from historical events (e.g., age at independence, 1958), or from an informant. The enumeration areas were used as survey sites. In each administrative region, one district was randomly selected. The list of the National Statistical Direction was used as a reference for the selecting of the number of enumeration areas. From this census, the capital and regional capitals were considered urban, and all other localities were considered rural. We pre-specified the number of enumeration areas to be selected per district at 30. In each enumeration area, 7 households were randomly selected using a systematic sampling method. Therefore, to achieve the required sample size, the total number of enumeration areas selected was 240 enumeration areas. In each enumeration area that was retained, the dwellings, households, and then the individuals were randomly selected as follows: the investigator was placed in the middle of the enumeration areas (neighbourhoods or villages) and randomly chose a direction. In this chosen direction, he entered in each dwelling. In the wells retained, he selected all the people known to have reached 60 years of age.

### Sample size

The sample size was calculated using the Schwartz formula, with a precision level of 5% and a type I error level of 5%. To assess the extent of malnutrition, the prevalence among elderly individuals in Guinea was estimated at 5.5% [22], with non-respondents expected to constitute approximately 60% of the sample size. Consequently, the survey encompassed a total of 1,698 subjects. It was multiplied by two, considering that a village corresponds to an enumeration area of individuals and selecting an enumeration area effect of 2. The minimum sample size was 210 per district and a total of 1680 individuals were needed for the country.

### Ethics approval and consent to participate

The study was approved by the National Ethics Committee for Health Research of Guinea (registered as #012/CNERS, 2021). Individuals diagnosed with hypertension were referred to health units. This study was conducted in accordance with the principles of the Declaration of Helsinki.

### Data collection

A structured interview using a standardized questionnaire was conducted by the investigators. Sixteen trained investigators final-year medical students) interviewed individuals in French. The questionnaire was translated into Susu, Pular, Maninka and Nguerze languages for participants who could not speak or understand French. The questionnaire included the socio-demographic data such as age, sex, residence (rural or urban), marital status (married or unmarried), education (none, primary and plus), previous occupation (craftsman / farmer, trader, government, unemployed), natural region (Forest Guinea, Lower Guinea, Middle Guinea, Upper Guinea), and monthly income (825 000FG≤, and ≥ 825 000FG). Data on medical characteristics such as history of diabetes (yes or no), extra salt (Intake was recorded when participants reported adding extra salt to their food, or when there was no intake reported) and body mass index (BMI) were also collected. BMI was calculated by dividing weight (kg) by the square of the height (m²). Underweight/normal was defined as BMI < 25, overweight was defined as 25 ≤ BMI < 30, and obesity as BMI of ≥ 30. Blood pressure was measured in the third sitting position for each participant. It was measured after the participant had rested quietly for 5 min, by a licensed nurse or a medical student using an OMRON electronic sphygmomanometer with a cuff. Two measurements were taken at 5-minute intervals, and the average of the last two measurements was used for the analysis. Hypertension was defined as systolic blood pressure ≥ 140 mm Hg and/or diastolic blood pressure ≥ 90 mm Hg, or current use of anti-hypertensive medication. Participants who reported the current use of antihypertensive medication were considered to have hypertension. Among known hypertensives, those receiving specific antihypertensive or on a specific diet at the time of the survey were classified as ‘‘treated hypertension’’. Among these subjects, those who had normal blood pressure on the day of the survey were classified as ‘‘controlled hypertension’’. Further classification of severity was undertaken with Grade 1 defined as ≥ 140/90 mm Hg, grade 2 hypertension defined as ≥ 160/100mmHg, and grade 3 hypertension as ≥ 180/110mmHg [23]. The pulse pressure was operationally defined as the difference between systolic blood pressure and diastolic blood pressure.

### Statistical analysis

All data analysis were weighted, using individual weights that were post-stratified based on the age structure of the Guinean national population in 2014 as the reference population. Age standardization was performed using the direct method. Confidence intervals (CIs) for prevalence were calculated. A logistic regression model with weighted data was constructed to identify independent predictors of hypertension. All significant factors in the univariable analysis (based on *P* < 0.20) were entered into a multivariable logistic regression model. Stepwise backward procedures were used to retain the final predictors of hypertension (*P* < 0.05). The quality of the final model was assessed by the Hosmer and Lemeshow goodness of fit test. Statistical interaction was tested between all predictors of hypertension and area in the final model. A model was constructed for the entire population and separately for each area. Data analysis was performed with STATA Version 16 (College Station, Tex.).

## Results

A total of 1698 subjects participated in this study, 63.5% of whom were men. The mean age was 71.6 ± 9.4 years and ranged from 60 to 115 years. Rural participants (mean age = 72.4 ± 9.7) were older (*p* < 0.001) than urban participants (70.0 ± 8.5). Participants were predominantly Maninka (32.6%), married (75.9%), unemployed (48.6%), and resided in Lower Guinea (38.2%). The distributions of the baseline sociodemographic characteristics of the study population by sex and location are summarized in Table 1.

**Table 1:** General characteristics of the study population by gender and area among the elderly in Guinea in 2021

Category		Sex						Area				Total age standardized (%)
		Total		Female		Male		Rural		Urban		(%)
		n	(%)	n	(%)	n	(%)	n	(%)	n	(%)	
Area	Rural	1097	(64.6)	355	(57.4)	742	(68.8)					(63.1)
	Urban	601	(35.4)	264	(42.6)	337	(31.2)					(36.9)
Sex	Female	619	(36.5)					355	(32.4)	264	(43.9)	(38.6)
	Male	1079	(63.5)					742	(67.6)	337	(56.1)	(61.4)
Age group. years	< 70	819	(48.2)	309	(49.9)	510	(47.3)	495	(45.1)	324	(53.9)	(55.6)
	≥ 70	879	(51.8)	310	(50.1)	569	(52.7)	602	(54.9)	277	(46.1)	(44.4)
Mean age. years (± s.d.)		71.6	(±9.4)	71.1	(±9.5	71.8	(±9.3	72.4	(±9.7)	70.0	(±8.5)	69.1(±8.3)
Marital status	Unmarried	409	(24.1)	350	(56.5)	59	(5.5)	236	(21.5)	173	(28.8)	(22.7)
	Married	1289	(75.9)	269	(43.5)	1020	(94.5)	861	(78.5)	428	(71.2)	(77.3)
Ethnic group	Fulani	487	(28.7)	179	(28.9)	308	(28.5)	363	(33.1)	124	(20.6)	(30.5)
	Maninka	553	(32.6)	176	(28.4)	377	(34.9)	403	(36.7)	150	(25.0)	(30.7)
	Nguerze	202	(11.9)	82	(13.2)	120	(11.1)	105	(9.6)	97	(16.1)	(11.9)
	Soussou	456	(26.9)	182	(29.4)	274	(25.4)	226	(20.6)	230	(38.3)	(26.9)
Previous occupation	Craftsman / Farmer	685	(40.3)	126	(20.4)	559	(51.8)	593	(54.1)	92	(15.3)	(42.0)
	Trader	119	(7.0)	63	(10.2)	56	(5.2)	42	(3.8)	77	(12.8)	(7.8)
	Government	68	(4.0)	9	(1.5)	59	(5.5)	20	(1.8)	48	(8.0)	(4.5)
	Jobless	826	(48.6)	421	(68.0)	405	(37.5)	442	(40.3)	384	(63.9)	(45.7)
Natural region	Forest Guinea	210	(12.4)	82	(13.2)	128	(11.9)	89	(8.1)	121	(20.1)	(11.8)
	Lower Guinea	648	(38.2)	270	(43.6)	378	(35.0)	321	(29.3)	327	(54.4)	(38.5)
	Middle Guinea	420	(24.7)	145	(23.4)	275	(25.5)	332	(30.3)	88	(14.6)	(26.7)
	Upper Guinea	420	(24.7)	122	(19.7)	298	(27.6)	355	(32.4)	65	(10.8)	(22.9)
Monthly income	< 825000FG	1535	(90.4)	607	(98.1)	928	(86.0)	1008	(91.9)	527	(87.7)	(89.8)
	≥ 825000FG	163	(9.6)	12	(1.9)	151	(14.0)	89	(8.1)	74	(12.3)	(10.2)
Extra salt	No intake	841	(49.5)	300	(48.5)	541	(50.1)	531	(48.4)	310	(51.6)	(50.6)
	Intake	857	(50.5)	319	(51.5)	538	(49.9)	566	(51.6)	291	(48.4)	(49.4)
History of diabetes	No	1595	(93.9)	578	(93.4)	1017	(94.3)	1054	(96.1)	541	(90.0)	(94.0)
	Yes	103	(6.1)	41	(6.6)	62	(5.7)	43	(3.9)	60	(10.0)	(6.0)
BMI. kg/m2	< 18.5	247	(14.5)	71	(11.5)	176	(16.3)	200	(18.2)	47	(7.8)	(12.9)
	18.5–24.9	1034	(60.9)	356	(57.5)	678	(62.8)	705	(64.3)	329	(54.7)	(60.6)
	25-29.9	321	(18.9)	126	(20.4)	195	(18.1)	160	(14.6)	161	(26.8)	(20.0)
	=>30	96	(5.7)	66	(10.7)	30	(2.8)	32	(2.9)	64	(10.6)	(6.4)
Mean BMI. kg/m2 (± s.d)		22.6	(±4.4)	23.6	(±5.0)	22.0	(±3.9)	21.8	(±3.9)	24.2	(±4.8)	(22.9±4.5)
Mean SBP. mmHg (± s.d)		146.1	(±25.5)	148.9	(±26.1)	144.5	(±25.1)	145.4	(±25.5)	147.3	(±25.5)	(145.6±25.3)
Mean DBP. mmHg (± s.d.)		86.9	(±14.5)	88.1	(±14.1)	86.3	(±14.7)	86.6	(±14.7)	87.5	(±14.2)	(87.0±14.5)

### Prevalence of hypertension

Of the 1,698 study participants in Guinea,1,059 (crude prevalence: 62.4%) had hypertension, resulting in an age- standardized prevalence of 61.4% (95% CI 61.3–61.6). The standardized prevalence of hypertension was higher in urban areas (61.6%, 95% CI 61.4–61.8) than in rural areas (61.3%, 95% CI 61.2–61.5). The prevalence of hypertension was significantly higher (*p* < 0.001 in women (65.2%, 95% CI 65.0–65.4) than in men (59.1%, 95% CI 58.9–59.3). The standardized prevalence of hypertension was 58.03% in participants aged 60–69 years, 65.63% in participants aged 70–79 years, 66.10% in participants aged 80–89 years, 64.56%in participants aged ≥ 90 years. The prevalence of hypertension in other major subgroups was 52.0% (Middle Guinea), 55.5% (Forest Guinea), 66.4% (Lower Guinea), and 67.1% (Upper Guinea) for natural region (*p* < 0.001); 60.2% (no diabetes) and 80.7% (diabetes) for diabetes status (*p* < 0.001); 55.1% (craftsman / farmer), 62.1% (trader), 65.5% (Government), and 66.7% (Jobless) for occupation (*P* < 0.001). In terms of BMI, the standardized prevalence of hypertension across underweight/normal, overweight, and obese was 58.9% (95% CI: 58.7–59.0), 66.4% (95% CI: 66.1–66.6) and 75.3% (95% CI: 74.9–75.9) respectively. Table 2 shows the prevalence of hypertension by category.

**Table 2:** Prevalence of hypertension, crude, and adjusted odds ratios for associated factors by area among the elderly in Guinea in 2021

Category		HTA (%)	95% CI	Univariate			Multivariate					
							Rural			Urban		
				OR	IC 95%	*p*	AOR	IC 95%	*p*	AOR	IC 95%	*p*
Area	Rural	61.3%	(61.2–61.5)	1.00	Reference	0.04						
	Urban	61.6%	(61.4–61.8)	1.01	(1.00–1.02)							
Sex	Female	65.2%	(65.0-65.4)	1.29	(1.28–1.30)		0.96	(0.94–0.98)		1.14	(1.12–1.17)	
	Male	59.1%	(58.9–59.3)	1.00	Reference	< 0.001	1.00	Reference	0.001	1.00	Reference	< 0.001
Natural region	Middle Guinea	52.0%	(51.8–52.2)	1.00	Reference	< 0.001	1.00	Reference	< 0.001	1.00	Reference	< 0.001
	Lower Guinea	66.4%	(66.2–66.6)	1.83	(1.80–1.85)		0.87	(0.85–0.89)		3.08	(2.97–3.20)	
	Forest Guinea	55.5%	(55.2–55.9)	1.15	(1.13–1.17)		2.14	(2.03–2.26)		0.88	(0.83–0.93)	
	Upper Guinea	67.1%	(66.8–67.3)	1.88	(1.85–1.91)		1.68	(1.63–1.73)		2.36	(2.25–2.48)	
Age group, years	< 70	58.0%	(57.9–58.2)	1.00	Reference	< 0.001	1.00	Reference	< 0.001	1.00	Reference	< 0.001
	≥ 70	65.7%	(65.5–65.9)	1.39	(1.37–1.40)		1.25	(1.23–1.27)		1.40	(1.37–1.43)	
Marital status	unmarried	67.7%	(67.5–68.0)	1.42	(1.40–1.47)	< 0.001	1.22	(1.19–1.25)		1.07	(1.05–1.10)	
	Married	59.6%	(59.5–59.7)	1.00	Reference		1.00	Reference	< 0.001	1.00	Reference	< 0.001
Ethnic group	Fulani	54.7%	(54.5–54.9)	1.00	Reference	< 0.001	1.00	Reference	< 0.001	1.00	Reference	< 0.001
	Maninka	65.2%	(65.0-65.4)	1.55	1.53–1.57		0.94	(0.92–0.97)		1.26	(1.21–1.31)	
	Nguerze	57.4%	(57.1–57.8)	1.12	1.09–1.14		0.50	(0.47–0.52)		1.71	(1.63–1.81)	
	Soussou	66.5%	(66.3–66.8)	1.65	1.62–1.67		1.32	(1.28–1.36)		0.88	(0.85–0.92)	
Previous occupation	Craftsman / Farmer	55.1%	(54.9–55.3)	1.00	Reference	< 0.001	1.00	Reference	< 0.001	1.00	Reference	< 0.001
	Trader	62.1%	(61.7–62.5)	1.34	(1.31–1.36)		1.78	(1.72–1.84)		1.29	(1.25–1.33)	
	Government	65.5%	(65.0-66.1)	1.55	(1.51–1.59)		1.22	(1.17–1.28)		1.66	(1.59–1.73)	
	Jobless	66.7%	(66.6–66.9)	1.64	(1.62–1.65)		1.67	(1.65–1.70)		1.29	(1.26–1.33)	
Monthly income	< 825000FG	62.1%	(61.9–62.2)	1.29	(1.26–1.31)		1.26	(1.23–1.29)		1.41	(1.37–1.46)	
	≥ 825000FG	56.0%	(55.6–56.4)	1.00	Reference	< 0.001	1.00	Reference	< 0.001	1.00	Reference	< 0.001
Extra salt	No intake	57.8	(57.6–58.0)	1.00	Reference	< 0.001	1.00	Reference	< 0.001	1.00	Reference	< 0.001
	Intake	65.2	(65.0-65.4)	1.37	(1.35–1.38)		1.16	(1.15–1.18)		1,05	(1,03 − 1,07)	
History of diabetes,	No	60.2%	(60.1–60.3)	1.00	Reference	< 0.001	1.00	Reference	< 0.001	1.00	Reference	< 0.001
	Yes	80.7%	(80.3–81.1)	2.77	(2.70–2.85)		1.61	(1.55–1.67)		3.59	(3.45–3.74)	
BMI, kg/m2	< 25	58.9%	(58.7–59.0)	1.00	Reference	< 0.001	1.00	Reference	< 0.001	1.00	Reference	< 0.001
	25–29,9	66.4%	(66.1–66.6)	1.38	(1.36–1.39)		1.74	(1.71–1.78)		1.19	(1.16–1.21)	
	=>30	75.3%	(74.9–75.8)	2.13	(2.08–2.18)		1.48	(1.41–1.53)		1.95	(1.88–2.02)	

### Detection, treatment, and control of hypertension

For all hypertensive subjects, only 53.3% (95% CI 53.1–53.4) were aware of their status. The prevalence of female subjects being aware of their diagnosis of hypertension (60.7: 95% CI 60.4–60.9) was significantly (*p* < 0.001) higher than that of male subjects (48.1: 95% CI 47.9–48.3).

The prevalence of urban subjects being aware of their diagnosis of hypertension (58.7: 95% CI 58.4–58.9) was significantly higher (*p* < 0.001) than that of rural subjects (50.1: 95% CI 49.9–50.3).

Among hypertensive who reported being aware of their hypertension, 49.6% (95% CI 49.3–49.8) were taking treatment. This prevalence of hypertensive treatment was significantly higher (*p* = 0.002) in male (49.9%) than in female (49.2%); significantly higher (*p* < 0.001) in urban subjects (51.1%) than in rural subjects (48.3%).

**Fig. 2:**
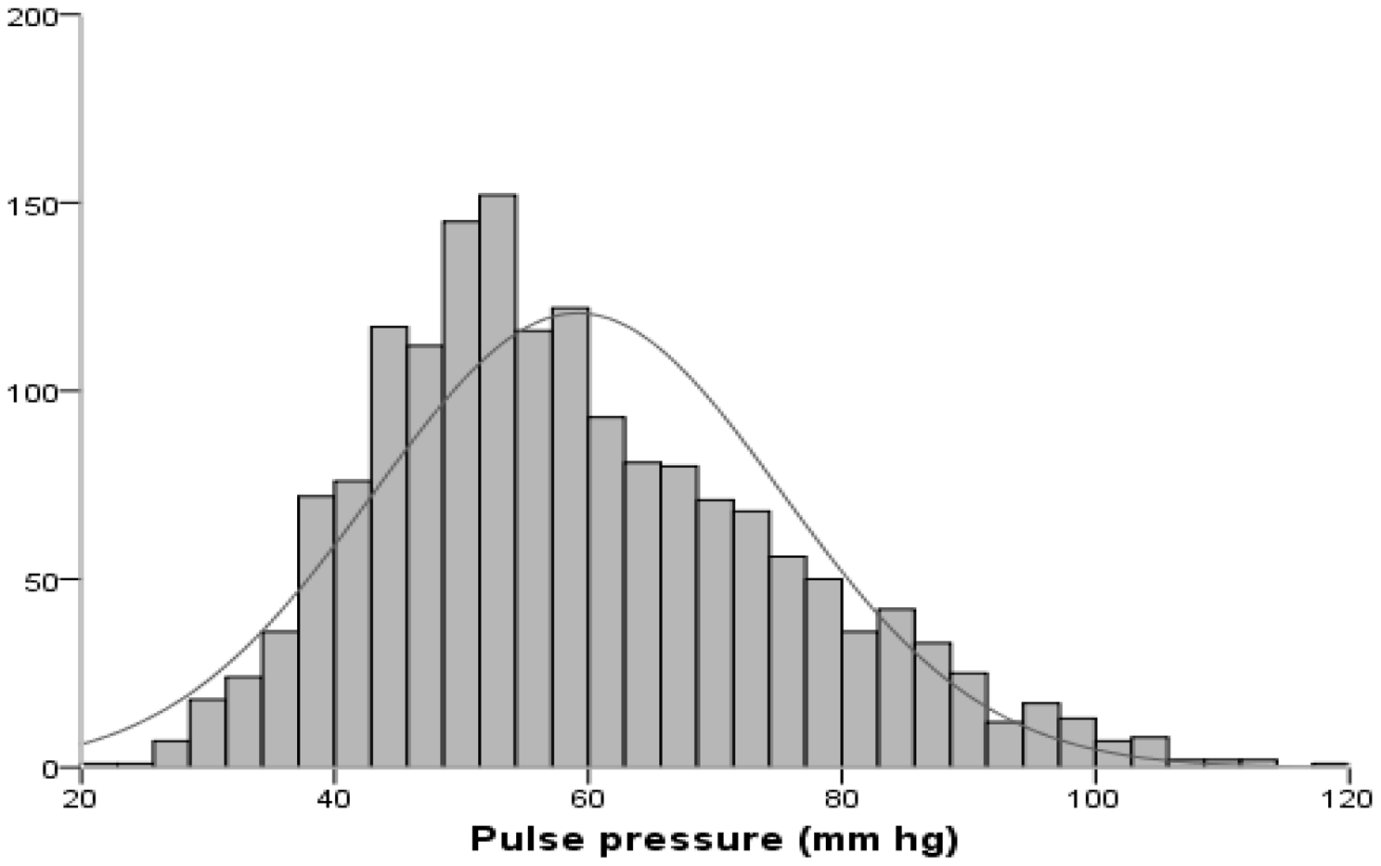
Histogram with normal distribution of pulse pressure among the elderly in Guinea in 2021

Among hypertensive individuals who were unaware of their hypertension, 51.1% exhibited systolo-diastolic hypertension, 40.1% had isolated systolic hypertension, and 8.8% had diastolic hypertension. Figure 2 depicts the histogram illustrating the normal distribution of pulse pressure among the elderly in Guinea in 2021. The average pulse pressure was significantly higher (*p* < 0.05) in hypertensive participants (65.92 ± 15.71 mmHg) compared to non-hypertensive individuals (47.96 ± 8.57 mmHg).

**Fig. 3:**
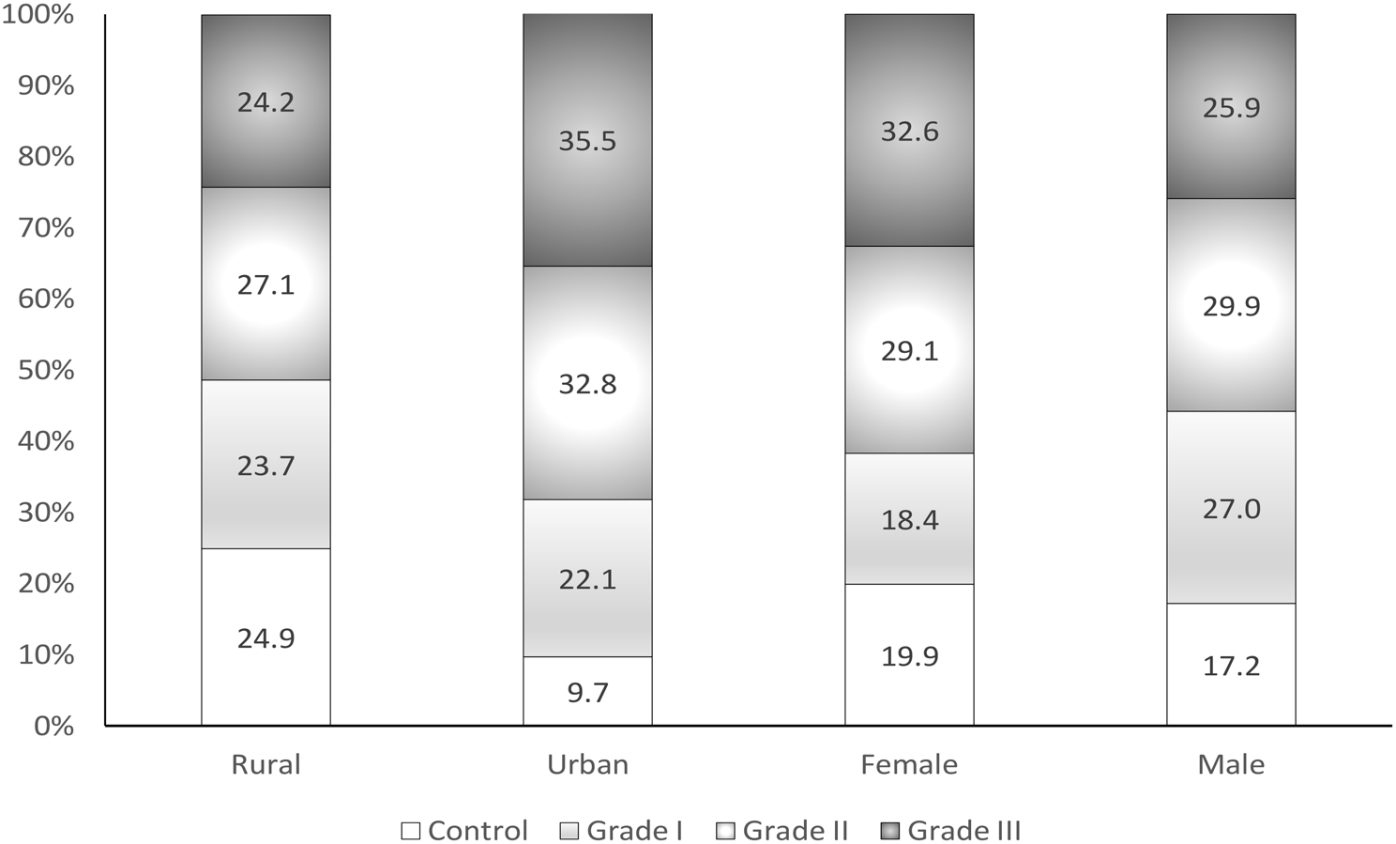
Percentage of controlled and grade of hypertension by sex and area among the elderly in Guinea in 2021

Among subjects who knew their diagnosis, only 18.5% (95% CI 18.2–18.7) had adequately controlled hypertension. The prevalence of adequately controlled hypertension in female subjects (19.9: 95% CI 19.6–20.3) was significantly (*p* < 0.001) higher than that in male subjects (17.2: 95% CI 16.9–17.6). The prevalence of hypertension adequately controlled in rural subjects (24.9: 95% CI 24.6–25.3) was significantly higher (*p* < 0.001) than that in urban subjects (9.7: 95% CI 9.4–10.0). The data illustrating the control and various grades of hypertension categorized by sex and geographical area are presented in Fig. 3.

Grade 2 hypertension was the most common grade of hypertension (29.5%), followed by grade 3 (29.0%). Overall, there was a significantly higher (*p* < 0.001) prevalence of grade 3 hypertension in urban subjects (35.5%) compared to rural subjects (24.2%). Grade 3 hypertension was higher (*p* < 0.001) in female subjects (32.6%) compared to male subjects (25.9%).

### Associated risk factors of hypertension

The univariate analysis showed that living in an urban area, female sex, residence region other than Middle Guinea, increasing age, being unmarried, being ethnic group other than Fulani, occupation other than Craftsman / Farmer, low monthly income, intake of extra salt, known diabetic and increasing of BMI were associated with an increased likelihood of hypertension (Table 2).

The interactions between area and sex, age group, marital status, natural region, ethnic group, intake of extra salt, BMI, and diabetes history were all significant (*p* ≤ 0.0001). The interaction terms between area and all other variables favoured doing the multivariate analysis stratifying by area.

In multivariate analyses, whatever the area of residence (rural and urban), increasing age (odds ratio in rural 1.25: 95% confidence interval 1.23–1.27 and odds ratio in urban 1.40: 95% confidence interval 1.37–1.43), being unmarried (1.22: 1.19–1.25 and 1.07: 1.05–1.10), working as trader (1.78: 1.72–1.84 and 1.29: 1.25–1.33), functionary (1.22: 1.17–1.28 and 1.66: 1.59–1.73), jobless (1.67: 1.65–1.70 and 1.29: 1.26–1.33), living in upper Guinea (1.68:1.63–1.73 and 2.36: 2.25–2.48), low monthly income (1.26: 1.23–1.29 and 1.41: 1.37–1.46), intake of extra salt (1.16: 1.15 1.18 and 1.05: 1.03–1.07), known diabetic (1.61: 1.55–1.67 and 3.59: 3.45–3.74), overweight (1.74: 1.71–1.78 and 1.19: 1.16–1.21), and obesity (1.48: 1.41–1.53 and 1.95: 1.88–2.02) increased the risk of hypertension with variable intensity.

In multivariate analyses for the sex, being female increased the risk of hypertension in urban area (1.14: 1.12–1.17) while in rural area, that decreased the risk (0.96: 0.94–0.98).

For the natural region, living in lower Guinea increased the risk of hypertension in urban area (3.08: 2.97–3.20) while in rural area, that decreased the risk (0.87: 0.85–0.89); living in forest Guinea increased the risk of hypertension in rural area (2.14: 2.03–2.26) while in urban area, that decreased the risk (0.88: 0.83–0.93); for the ethnic group, being Maninka increased the risk of hypertension in urban area (1.26: 1.21–1.31), while in rural area, that decreased the risk (0.94: 0.92–0.97); being Nguerze increased the risk of hypertension in urban area (1.71: 1.63–1.81), while in rural area, that decreased the risk (0.50: 0.47–0.52); being Soussou increased the risk of hypertension in rural area (1.14: 1.12–1.17) while in urban area, that decreased the risk (0.88: 0.85–0.92).

## Discussion

To our knowledge, this study is the first investigation of the prevalence and associated factors of hypertension in a large representative sample of the elderly Guinean population.

We observed a high prevalence of hypertension (61.4%) that was driven principally by BMI and age. As people age, they are more likely to develop hypertension, and there is a biological basis for this phenomenon such as the changes in the structure and function of the arteries [24]. We found a lower prevalence of hypertension in rural areas compared to urban areas. However, the traditional rural-urban gradient observed in several African studies has been decreasing over the last 20 years [11, 25, 26].

In Africa, the elderly, who are relatively most affected by hypertension, are often neglected as a public health challenge [27]. Compared with Guinean younger adults [20], older individuals are at least twice more likely to develop hypertension. The prevalence of hypertension (61.4%) observed in the present study is similar to that reported by studies conducted in subjects aged ≥ 65 years in Nigeria [28], in Central Africa [10] and Cameroon in subjects aged ≥ 50 years [29], that reported rates of 62.2%, 61.1% and 57.3%, respectively. However, the prevalence mentioned here is higher than the prevalence report in Ghana (47.8%) in adults aged > 60 years [30]. In contrast, higher the prevalence levels were indicated in Burkina-Faso (82%), and Tanzania (78.5%) [31, 32]. In a systematic review, Bosu [11] estimated the prevalence of hypertension of the adults aged > 50 years in Africa ranged from 22.3 to 90.0% from the individual studies while the combined overall prevalence was 57.0% (95% CI 52%-61%). Comparison of the hypertension rates reported here with other recent studies from Africa is difficult by differences in methodology (the 60 and 65 year thresholds have been criticized as arbitrary and irrelevant for the African setting) and by dietary habits in the countries [11, 19]. The high prevalence of hypertension in elderly populations in Guinean in both rural and urban areas suggests more attention is warranted regarding the cardiovascular health of this group. Comprehensive control measures are urgently needed to control this epidemic by public health authorities. The goal of implementing these strategies would be to reduce the overall burden of the condition or disease on individuals, families, and society. In this study, only 53.3% of hypertensive participants were aware of their condition. Only 49.6% among them took antihypertensive treatments, and among them, 18.5% had their blood pressure controlled. In Central Africa [10], among hypertensive people, 46.7% were aware of their condition and 17.3% were treated, but 23.8% had their hypertension controlled. Our study showed that overall management of hypertension is poor in urban participants compared to rural. Further reports show that even with a relatively lower prevalence of hypertension among rural dwellers, the detection and overall management are poor in comparison to urban dwellers [25]. In general, in older adults in Africa, many cases of hypertension are detected late, treatments rarely follow standard guidelines, and the costs of medications are generally high [13].

The findings of our study, suggest the need to improve awareness and access to health services for the screening and treatment of hypertension in Guinea. To achieve this, the authorities of Guinea must follow the recent recommendations which suggest all African countries to adopt several solutions to achieve better hypertension management [13]. The following three goals should be achieved in Africa by 2030: (a) 80% of adults with high blood pressure in Africa are diagnosed; (b) 80% of diagnosed hypertensives, that is, 64% of all hypertensives, are treated; and (c) 80% of treated hypertensive patients are controlled [13].

Our results confirm some previously observed associations, such as increasing age [10, 19], occupation [10], monthly income [33], intake of extra salt [34], advancing BMI [19, 34, 35] and diabetes status [36, 37] being associated with an increased prevalence of hypertension. Compared to married individuals, being unmarried was independently associated with hypertension. This may be because married people have a better satisfaction with life [38]. In analysis by area, we found an inconsistent relationship between sex, ethnic group, and natural region with hypertension in the current study. These inconsistent differences appear to be declining probably due to nutritional transition [39, 40]. In a systematic review, Bosu [17] found consistent determinants of hypertension (overweight/obesity and history of stroke), less consistent but frequent determinants (including older age group, female sex and urban residence), inconsistent determinants (education, wealth index, alcohol consumption, physical activity, etc.) and insignificant covariates (marital status, having health insurance).

Our findings suggest health authorities should prioritize policies and develop programmes to support the improvement of the cardiovascular health of older population in Guinea. This programme must work to promote active ageing. Developing primary prevention of hypertension (hygienic-dietary measures, and physical exercise) and improving therapeutic care must be central to this programme. Weight, intake of extra salt, poverty reduction should be an important component of such programmes [25, 41]. Indeed, Aburto et al., showed that lowering salt intake to less than 1200 mg per day was safe and beneficial [41]. Further research is urgently needed on hypertension in the elderly. Longitudinal studies which use consistent study methods can fill in some of the data gaps including the trends and determinants of hypertension.

### Limitations

We used a cross-sectional design and as such, we are unable to consider trends over time. Behavioural factors were studied based on subject declarations, and therefore reporting bias may have been introduced. However, they were minimized by the fact that the investigated factors were non-stigmatizing and of short duration of involvement. Weight was measured in casual clothes without shoes. Asking people to undress would not have been widely acceptable in this elderly population. Nevertheless, most people wore only light clothing and any bias on BMI is likely to be relatively small. Finally, other potential sources of bias include the high rate of men in the sample (> 50%) certainly due to their greater presence in households at the time of the survey teams’ visit. The use of a door-to door survey, the large sample size, and a health professional for blood pressure measurement are strengths of our study.

## Conclusions

This large multicentre study has shown a high prevalence of hypertension, low awareness, and low control of hypertension in older in Guinea. Our study emphasises the challenges of non-communicable diseases management in sub-Saharan Africa from the perspectives of the difficulty in accessing healthcare in a resource-poor country. The detecting and treating hypertension in the elderly population that is rapidly growing in SSA can be highly beneficial.

## Data Availability

The datasets used and/or analysed during the current study are available from the corresponding author on reasonable request.
